# Supplementary material for: Performance inflation in junior tennis: Longitudinal analysis and Bayesian forecasting of ranking thresholds, efficiency, and access equity
Source: PLoS One. 2026 Apr 21;21(4):e0345725. doi: 10.1371/journal.pone.0345725 (PMC13098973; doi:10.1371/journal.pone.0345725)
Supplement: Attachment — Submitted filename: Response to Reviewers.docx [file pone.0345725.s013.docx]

**Performance inflation in junior tennis: longitudinal analysis and Bayesian forecasting of ranking thresholds, efficiency, and access equity**

**Journal Requirements:**

1. Please ensure that your manuscript meets PLOS ONE's style requirements, including those for file naming. The PLOS ONE style templates can be found at
[https://journals.plos.org/plosone/s/file?id=wjVg/PLOSOne_formatting_sample_main_body.pdf](https://track.editorialmanager.com/CL0/https:%2F%2Fjournals.plos.org%2Fplosone%2Fs%2Ffile%3Fid=wjVg%2FPLOSOne_formatting_sample_main_body.pdf/1/010f019c9e082813-6a2226b3-fce3-46fe-b396-ee931e6bb8cc-000000/QDaD4HEfIS6c9f5HvNy8Fsh6poV2FgApUDZXCJDEILU=252) and [https://journals.plos.org/plosone/s/file?id=ba62/PLOSOne_formatting_sample_title_authors_affiliations.pdf](https://track.editorialmanager.com/CL0/https:%2F%2Fjournals.plos.org%2Fplosone%2Fs%2Ffile%3Fid=ba62%2FPLOSOne_formatting_sample_title_authors_affiliations.pdf/1/010f019c9e082813-6a2226b3-fce3-46fe-b396-ee931e6bb8cc-000000/s49cOpaPLrYCbRWUbk8A56MLVxAkkxGIebVMdNjYrHU=252)

**Response:**

The manuscript has been carefully reviewed to ensure full compliance with PLOS ONE formatting and style requirements. File naming conventions, as well as formatting of the main manuscript.

2. Please note that PLOS One has specific guidelines on code sharing for submissions in which author-generated code underpins the findings in the manuscript. In these cases, we expect all author-generated code to be made available without restrictions upon publication of the work. Please review our guidelines at <https://journals.plos.org/plosone/s/materials-and-software-sharing#loc-sharing-code> and ensure that your code is shared in a way that follows best practice and facilitates reproducibility and reuse.

The journal’s guidelines regarding code sharing have been carefully reviewed and addressed. To facilitate transparency and reproducibility, a reproducible analysis script documenting the main data-processing steps, statistical modelling procedures, and forecasting workflow has been provided in the Supplementary Materials (S3 File). This script represents a cleaned and structured version of the analytical workflow prepared for reproducibility purposes. In addition, the empirical threshold dataset used as the primary input for the forecasting analyses has been made available in S8 Table. Together, these materials allow the analytical procedures underlying the reported results to be reproduced and comply with the PLOS ONE requirements for data and code availability.

3. We note you have included a table to which you do not refer in the text of your manuscript. Please ensure that you refer to Table 3 in your text; if accepted, production will need this reference to link the reader to the Table.

**Response:**
Thank you for this observation. An explicit reference to Table 3 has now been inserted into the section *“Tournament participation and point-accumulation efficiency”*, specifically within the paragraph reporting the correlation analysis between Total Ranking Points (TRP) and Points per Event (PPE). The manuscript has been revised accordingly to ensure appropriate cross-referencing and to comply fully with PLOS ONE production requirements.

4. We note that there is identifying data in the Supporting Information file <S1 File_Dataset_WTTJ_Male_2004-2024.pdf, S1 Table_Thresholds_TRP_2004_2024, S2 File_ITF_Tournaments_2004-2024.pdf, S2 Table_Thresholds_PPE_2004_2024.pdf >. Due to the inclusion of these potentially identifying data, we have removed this file from your file inventory. Prior to sharing human research participant data, authors should consult with an ethics committee to ensure data are shared in accordance with participant consent and all applicable local laws.

Data sharing should never compromise participant privacy. It is therefore not appropriate to publicly share personally identifiable data on human research participants. The following are examples of data that should not be shared:

-Name, initials, physical address
-Ages more specific than whole numbers
-Internet protocol (IP) address
-Specific dates (birth dates, death dates, examination dates, etc.)
-Contact information such as phone number or email address
-Location data
-ID numbers that seem specific (long numbers, include initials, titled “Hospital ID”) rather than random (small numbers in numerical order)

Data that are not directly identifying may also be inappropriate to share, as in combination they can become identifying. For example, data collected from a small group of participants, vulnerable populations, or private groups should not be shared if they involve indirect identifiers (such as sex, ethnicity, location, etc.) that may risk the identification of study participants.

Additional guidance on preparing raw data for publication can be found in our Data Policy (<https://journals.plos.org/plosone/s/data-availability#loc-human-research-participant-data-and-other-sensitive-data>) and in the following article: <http://www.bmj.com/content/340/bmj.c181.long>.

Please remove or anonymize all personal information (<specific identifying information in file to be removed>), ensure that the data shared are in accordance with participant consent, and re-upload a fully anonymized data set. Please note that spreadsheet columns with personal information must be removed and not hidden as all hidden columns will appear in the published file.

**Response:**

The editor’s concern regarding potentially identifiable information has been carefully addressed. The dataset used in this study originates from publicly available records provided by the International Tennis Federation (ITF) (https://www.itftennis.com/). As stated in the Methods section (Study design & data source), all data were processed and anonymised prior to analysis. In accordance with the journal’s data-sharing policy, the Supporting Information files have been revised and re-uploaded in a fully anonymised format. All potentially identifying information has been removed from the shared dataset. Variables retained in the shared files include only those necessary to reproduce the analyses presented in the manuscript (e.g., year of observation, year of birth, and country). These variables were preserved because they constitute essential analytical inputs used in the statistical procedures reported in the study. The revised dataset therefore complies with the journal’s requirements regarding participant privacy while preserving the information required for reproducibility of the reported analyses.

5. If the reviewer comments include a recommendation to cite specific previously published works, please review and evaluate these publications to determine whether they are relevant and should be cited. There is no requirement to cite these works unless the editor has indicated otherwise.

**Response:**

The publications suggested by the reviewer were carefully examined and considered during the revision process. These references served primarily as relevant background material that informed the methodological evaluation and the subsequent revision decisions. However, after careful consideration, they were not incorporated into the manuscript, as they were used mainly as supporting resources rather than as directly cited literature. Nevertheless, the reviewer’s suggestions and the provided references are appreciated, as they contributed to the critical assessment of the methodological approach and the overall revision process.

**Reviewers' comments (reviewer #1)**

The manuscript presents original research on longitudinal trends in junior tennis performance using a large dataset (2004-2024; n = 8082 players). The topic is relevant, and the combination of descriptive analysis, regression modeling, and forecasting is potentially valuable. The manuscript is generally well written and intelligible, and the research question is clearly stated.
However, when evaluated the requirements that “experiments, statistics, and other analyses are performed to a high technical standard” and that “conclusions are supported by the data”, there are some concerns regarding the statistical validity and reporting transparency.

**Response:**

The reviewer’s careful evaluation of the manuscript and the time devoted to the review process are greatly appreciated. The constructive comments and suggestions provided have been very valuable for improving the statistical clarity, reporting transparency, and overall methodological rigor of the manuscript. All comments have been carefully considered, and corresponding revisions have been implemented throughout the manuscript to address the concerns raised.

(1) Logistic regression. The use of binary logistic regression to estimate percentile thresholds is not the best choice for the goal of the paper. Percentiles are deterministic properties of the data distribution and can be computed directly. Modeling them via logistic regression introduces unnecessary complexity and potential bias without clear inferential benefit. A direct quantile-based approach or quantile regression would be more appropriate.

**Response:**

This point is well taken. Percentile thresholds are deterministic properties of the empirical performance distributions and can be computed directly. The original modelling step was intended to provide a smoothed threshold-identification function based on percentile membership rather than to redefine the empirical percentiles themselves.

To address the concern regarding unnecessary modelling complexity, a dedicated robustness analysis was conducted using directly computed annual empirical percentiles (P90, P75, P50) for both TRP and PPE (2004–2024). Agreement between empirical and logistic-derived cut-offs was quantified across years using mean absolute error (MAE), root mean square error (RMSE), maximum absolute deviation, and Pearson correlations.

The two approaches demonstrated very high agreement for PPE (r = .996/.981/.955 for P50/P75/P90) and for TRP in the mid-range percentiles (r = .999–1.000 for P50–P75). Lower agreement was observed for the TRP P90 series (r = .762), indicating that model-based cut-offs are more sensitive to year-specific shifts in the upper tail of the distribution. However, despite these absolute deviations, the directionality and long-term trajectory of the threshold series remained consistent, and re-forecasting based on empirical percentiles did not materially alter projected growth patterns. Importantly, re-estimation and forecasting based on empirical percentiles produced materially consistent longitudinal trajectories and did not alter the substantive conclusions regarding temporal inflation patterns and projected threshold growth. The manuscript and Supplementary Materials have been revised accordingly.

(2) Coherence of the statistical analysis. The workflow of the statistical analysis raises some concerns about internal coherence: Percentiles are first computed from the data, then used to define binary outcomes, and subsequently re-estimated via regression models whose outputs are later forecasted. This implies that the reported trends are, in part, artefacts of the modeling strategy rather than independent empirical findings. Why don’t use directly the empirical percentiles to make the predictions?

**Response:**

The concern regarding internal coherence is acknowledged. Percentile thresholds are deterministic features of the empirical annual distributions; therefore, direct empirical percentiles (P90, P75, P50) are the most parsimonious basis for longitudinal threshold tracking and forecasting. In the revised analysis, forecasting is performed on the directly computed annual empirical thresholds for both TRP and PPE.

Binary logistic regression is retained as a complementary threshold-identification framework to characterise percentile membership probabilistically and to generate model-based cut-offs for sensitivity comparison rather than as the primary basis for forecasting. Agreement between empirical and model-based cut-offs is quantified (MAE, RMSE, maximum absolute deviation, and Pearson correlations), and forecasts based on empirical thresholds yield materially consistent trajectories and projected growth patterns. The Methods and Supplementary Materials have been revised to clarify this analytical hierarchy and to avoid any ambiguity regarding circularity

(3) Interpretation in the presence of collinearity. The interpretation of TRP and PPE as complementary dimensions of performance is not fully supported statistically. The extremely high correlation between them (ρ ̂=0.947) is largely driven by their mathematical relationship, since PPE is derived from TRP. As a result, the claim that PPE captures an independent efficiency construct is not proven. This affects the validity of the conclusions.

**Response:**

This comment is well taken. The very high correlation between Total Ranking Points (TRP) and Points per Event (PPE) is structurally expected, as PPE is mathematically derived from TRP (PPE = TRP / Events). PPE therefore cannot be interpreted as a statistically independent construct. The manuscript has been revised accordingly to eliminate any wording that could imply independence between these indicators.

The analytical intention was not to present PPE as an independent latent dimension of performance, but rather as an efficiency-normalised transformation of cumulative ranking output. Whereas TRP reflects absolute point accumulation—strongly influenced by tournament participation opportunities—PPE expresses the rate of point acquisition conditional on event exposure. In this sense, PPE does not constitute an independent construct, but an alternative operational perspective on how ranking points are accumulated across differing participation densities.

To address the concern, the Abstract, Results, Discussion, and Conclusion sections were revised. Terms such as “independent” or “distinct mechanisms” were removed or reformulated. Interpretations were clarified to emphasise that TRP and PPE represent structurally related indicators, with PPE interpreted strictly as an efficiency-normalised metric derived from cumulative scoring.

These revisions ensure that the interpretation is statistically coherent in the presence of collinearity and that the conclusions are framed in a conceptually precise and methodologically defensible manner.

(4) Forecasting validation. Regarding the forecasting, although the use of a Bayesian Prophet model is acceptable, the absence of any form of model validation (e.g., out-of-sample testing, cross-validation, or predictive error metrics) is a major limitation. Without validation, it is not possible to assess whether the forecasts are reliable or simply extrapolations of past trends. For instance, you could use the last years of your sample to test your model.

**Response:**

A formal time-series validation procedure was incorporated to evaluate the predictive performance of the Bayesian Prophet forecasting framework applied to the empirically derived percentile-based time series (TRP and PPE at P90, P75, and P50). Rolling-origin (walk-forward) cross-validation was implemented with a three-year forecast horizon and a one-year step size. The model was repeatedly re-estimated on expanding training windows (minimum training length ≈10 years), and out-of-sample predictive accuracy was quantified using mean absolute error (MAE), root mean square error (RMSE), and mean absolute percentage error (MAPE). COVID-affected years (2020–2021) were excluded from both model estimation and validation to avoid distortion from exogenous scheduling disruptions.

Across the six percentile series, cross-validated point-forecast errors were of comparable magnitude, with MAPE values ranging from 21.0% to 25.2% (PPE: MAE 5.33–14.1; TRP: MAE 158–293; N_test = 18 forecasts per series). These results were incorporated into the revised manuscript and Supplementary Materials to document formal out-of-sample predictive performance.

In addition, empirical coverage of the nominal 95% predictive intervals was assessed. Observed coverage ranged approximately between 28% and 33% across series. This deviation from nominal coverage likely reflects the short length of annual time series, structural changepoints, and the limited number of validation windows available. Accordingly, interval estimates were interpreted cautiously, and the revised manuscript places primary inferential emphasis on cross-validated point-forecast accuracy rather than interval calibration.

Collectively, this validation framework demonstrates that the forecasting results are supported by systematic out-of-sample evaluation rather than relying solely on in-sample trend extrapolation.

(5) COVID-19 pandemic years. The treatment of COVID-19 years introduces inconsistencies: the years of the pandemic (2020-2021) are excluded from forecasting but included in descriptive and regression analyses. While this decision may be reasonable, a sensitivity analysis would be necessary to demonstrate that conclusions are robust to this choice.

**Response:**

The exclusion of COVID-affected seasons (2020–2021) from the forecasting models, while retaining them in descriptive and regression analyses, was acknowledged as requiring an explicit robustness assessment. A formal sensitivity analysis was therefore conducted in which the Bayesian Prophet models were re-estimated using the full empirical percentile time series, including the years 2020–2021 (TRP and PPE at P90, P75, and P50), under the same model specification as in the primary analysis.

Inclusion of the pandemic years resulted in systematically lower point forecasts for the 2025–2029 horizon across all six percentile series, indicating that the COVID-related disruption introduced a structural downward shift that propagated into subsequent projected trends (e.g., PPE P50: 2025 forecast 20.5 when COVID years were included vs. 29.1 when excluded). Furthermore, rolling-origin cross-validation revealed substantially degraded out-of-sample predictive performance when COVID years were incorporated, consistent with the presence of an anomalous structural shock not representative of post-pandemic competitive dynamics.

In light of these findings, the exclusion of 2020–2021 was retained as the primary forecasting specification. The results of the sensitivity analysis, including forecast comparisons and predictive error metrics, are now reported in the Supplementary Materials to explicitly document the impact of alternative treatment of the pandemic period.

(6) Skewness. The variables involved in the analysis exhibit strong skewness and heavy tails (see, for example, figure 2), yet these features are not fully addressed in the modeling strategy. The absence of transformations, robustness checks, or validation of the model raises concerns about the stability of the results.

**Response:**

A robustness check addressing distributional skewness and heavy tails was added by re-estimating the Bayesian Prophet forecasting models on log-transformed empirical percentile series (log(y)) and then back-transforming the point forecasts to the original scale for comparability. The log-specification used the same Prophet configuration as the main forecasting analysis, with forecasts generated for 2025–2029 for each series (TRP and PPE at P90, P75, and P50).

The log-based specification produced systematically lower back-transformed point forecasts relative to the original-scale models across all series, with mean differences ranging from −46% to −76% (maximum absolute differences: 48%–76%). The largest discrepancies were observed for TRP_P50 (mean −72.8%, max 76.0%) and PPE_P50 (mean −63.1%, max 66.7%). These results indicate that a log-scale Prophet specification imposes a markedly different growth geometry and yields forecasts that are not numerically commensurate with the original-scale specification in this application. Consequently, the primary analyses were retained on the original scale, and the log-based results are reported as a sensitivity analysis to transparently document the impact of alternative variance-stabilising transformations on forecast magnitudes (in supplementary materials).

(7) Longitudinal data. The longitudinal nature of the dataset is not adequately accounted for. Observations appear to be treated as independent, despite repeated measurements across years. Instead of fitting separate models for each year, the author should be considered to fit a single model for each outcome.

**Response:**

Thank you for this observation. The present study does not analyse repeated measurements of individual players over time; rather, the unit of analysis is the annual distribution of performance outcomes across the full population of ranked junior players. For each season (2004–2024), percentile-based performance thresholds (P90, P75, P50) were computed from the cross-sectional distribution of year-end Total Ranking Points (TRP) and Points per Event (PPE). These percentile thresholds represent aggregated benchmarks describing the structure of the ITF junior ranking system in a given year rather than longitudinal trajectories of individual athletes.

Consequently, the analytical dataset used for forecasting consists of six aggregated time series (TRP_P90, TRP_P75, TRP_P50, PPE_P90, PPE_P75, PPE_P50), each representing a single annual performance threshold observed once per year. These series therefore constitute time-series observations of evolving competitive benchmarks rather than repeated player-level measurements. The forecasting models were accordingly specified as time-series models applied to these aggregated percentile thresholds.

To clarify this design choice, additional explanatory text has been inserted into the Methods and discussion section describing the aggregation procedure and explicitly defining the unit of analysis as annual percentile-based performance thresholds derived from cross-sectional player distributions.

(8) Interpretation. Several conclusions, particularly those regarding “performance inflation”, being driven by distinct mechanisms (structural vs efficiency-based), are not fully supported by the statistical evidence, given the strong dependence/correlation between TRP and PPE and the limitations of the modeling approach. The conclusions should therefore be presented more cautiously together with the abovementioned limitations.

Reporting and transparency (EQUATOR / PLOS ONE standards)
I strongly recommend that the author look at some of the EQUATOR network's (https://www.equator-network.org/) publishing guidelines, which, although mainly focused on the biomedical field, may help in some aspects of this article.

**Response:**

The reviewer’s insightful comment regarding the interpretation of the relationship between cumulative ranking points (TRP) and efficiency-based scoring (PPE) is appreciated. The strong correlation between these indicators (ρ = .95) is acknowledged, and it is recognised that this relationship requires cautious interpretation and does not support a fully independent interpretation of the mechanisms underlying performance inflation. To address this concern, several modifications have been implemented throughout the manuscript:

1. Interpretation has been softened in the Abstract, Discussion, and Conclusion sections to avoid causal language implying that performance inflation is driven by two independent mechanisms. Instead, TRP and PPE are now explicitly described as complementary operational perspectives on the same competitive process (cumulative point accumulation versus efficiency conditional on tournament exposure).
2. The structural dependence between TRP and PPE has been explicitly acknowledged. PPE is mathematically derived from ranking points and participation volume and should therefore be interpreted as an efficiency-normalised transformation of cumulative performance rather than a statistically independent construct.
3. A new paragraph has been added to the Limitations section, explicitly addressing the strong correlation between the two indicators and clarifying that interpretations regarding structural versus efficiency-based dynamics should be viewed as descriptive rather than causal explanations.

(9) STROBE. According to STROBE Statement E&E [1], key elements are insufficiently detailed: missing data handling, potential sources of bias, or data preprocessing steps, etc. This limits reproducibility and transparency.

**Response:**

The reviewer’s emphasis on transparent reporting in accordance with STROBE recommendations is appreciated. To improve reproducibility and methodological clarity, several additions have been made to the *Methods* section.

Specifically, explicit descriptions have now been included regarding:

1. Data preprocessing procedures, including screening of publicly available ITF records and the treatment of derived variables (TRP, PPE, and age).
2. Handling of missing data, clarifying that missing values were minimal due to the standardized structure of the ITF year-end ranking records and that no imputation procedures were applied.
3. Potential sources of bias, including:
   – selection bias related to the use of year-end ranking lists,
   – structural changes in tournament availability across years,
   – disruptions caused by the COVID-19 pandemic.

Additionally, it has been clarified that pandemic-affected seasons were retained for descriptive transparency but treated separately in forecasting analyses.

(10) TRIPOD. According to TRIPOD Statement E&E [2], the manuscript falls short of expectations: There is no clear description of model validation, no assessment of predictive performance, and limited detail on model specification.

**Response:**

The reviewer’s suggestion to strengthen the reporting of the predictive modelling framework in accordance with TRIPOD recommendations is appreciated.

To address this issue, the manuscript has been revised in several ways:

1. Model specification has been clarified in the *Forecasting Procedures* subsection, including the configuration of the Bayesian Prophet model (changepoint settings, prior regularisation, and MCMC sampling parameters). The description of model validation procedures has been expanded, explicitly stating that rolling-origin cross-validation was used to evaluate predictive robustness across sequential training windows and forecast horizons. Additional information has been added describing predictive performance metrics, including Mean Absolute Error (MAE), Root Mean Square Error (RMSE), Mean Absolute Percentage Error (MAPE), and empirical coverage of prediction intervals.
2. It has been clarified that empirically derived percentile thresholds constitute the primary forecasting inputs, whereas logistic regression–derived thresholds are used only as a sensitivity and robustness check, with detailed results reported in the Supplementary Materials.

(11) Data & Code availability. All the reporting guidelines recommend to share the data and the analysis code. The author mentions that the data is available. However, the format used to share the data (PDF in the supplementary material) is not appropriate because it does not comply with the FAIR principles (https://www.go-fair.org/fair-principles/). Furthermore, the analysis code has not been shared either. For full reproducibility, it would be desirable to provide code or more detailed information on data processing and model implementation.

Response:

The concern regarding FAIR compliance is acknowledged. The Supporting Information has been revised to replace the previously provided PDF-formatted dataset with machine-readable files (CSV) accompanied by a data dictionary/codebook. In addition, the complete analysis workflow has been shared as a reproducible R project, including a single entry-point script to regenerate all tables and figures reported in the manuscript, as well as an environment lockfile to ensure package-version reproducibility.

The shared materials contain only de-identified and/or aggregated variables required to reproduce the reported results, thereby maintaining participant privacy while enabling full computational reproducibility.

(12) The use of an arbitrary threshold (R^2>0.20) to retain logistic models is not justified. See, for example the article of Heinze & Dunker [3].

(13) The interpretation of Nagelkerke’s R^2 as “explanatory power” should be avoided: "Power" has a specific meaning in statistics and should not be used in other contexts that could lead to ambiguity.

Response (to 12 and 13 note):

The reviewer’s comment regarding the use of an arbitrary threshold for Nagelkerke’s R² is appreciated. In the revised manuscript, the previously applied criterion (R² > 0.20) has been removed. Nagelkerke’s R² is now reported solely as a descriptive measure of model fit rather than as a decision criterion for retaining models.

Additionally, the terminology referring to “explanatory power” has been revised to avoid ambiguity, as the term “power” has a specific meaning in statistical inference. The manuscript now refers to Nagelkerke’s R² as an indicator of model fit. These changes align the statistical interpretation with current recommendations for regression modelling and avoid the use of arbitrary thresholds in model evaluation.

(14) In several parts of the manuscript, the notation of population parameters is confused with their estimation; for example, β_0,β_1,ρ should be replaced by \hat{β}_0, \hat{β}_1, \hat{ρ}.

**Response:**

The reviewer’s comment regarding the distinction between population parameters and their sample estimates is appreciated. In the revised manuscript, the notation has been corrected throughout the text to distinguish between theoretical population parameters and estimated quantities. Specifically, regression coefficients are now denoted as $\backslash hat\left\{ \backslash beta \right\}_{0}$ and $\backslash hat\left\{ \backslash beta \right\}_{1}$, where appropriate.

(15) Some typos:
-P7L149. An space before “held” is missing
-P8L178 & P8L180. These two consecutive sentences are redundant.

**Response:**

Thank you for carefully identifying these typographical issues. The missing space has been corrected, and the redundant sentences have been removed in the revised manuscript.
